# Enhancing Emergency Department Safety: Implementation of a Dedicated Chest Drain Trolley to Optimize Care for Pneumothorax Patients

**DOI:** 10.7759/cureus.76489

**Published:** 2024-12-27

**Authors:** Moustafa Elramlawy, Sunil Parmar, Adelaide Yue, Hany Abdelmasih, Michelle Denham, Annie Donaghy, Arif Ahmad, Basma Elshantory

**Affiliations:** 1 Emergency Department, Stoke Mandeville Hospital, Aylesbury, GBR; 2 School of Clinical Medicine, University of Cambridge, Cambridge, GBR; 3 Acute Medicine Department, Stoke Mandeville Hospital, Aylesbury, GBR; 4 Internal Medicine Department, Ain Shams University Demerdash Hospital, Cairo, EGY

**Keywords:** chest drain, chest drain trolley, optimum timing of chest drain insertion, patient-centred care, quality improvement projects

## Abstract

Delays in accessing chest drain equipment in the Emergency Department (ED) posed significant risks to patient safety, particularly for those with life-threatening pneumothorax. This quality improvement project (QIP) aimed to reduce these delays by implementing a dedicated chest drain trolley using the Plan-Do-Study-Act (PDSA) methodology. Surveys and simulations identified key issues, including equipment inaccessibility and staff unfamiliarity, with baseline preparation times exceeding 20 minutes. Iterative changes, such as labeling drawers, reorganizing supplies, and creating checklists, reduced preparation times to 6-7 minutes. Real-case feedback highlighted improved efficiency, staff confidence, and patient safety. This project demonstrates the value of structured quality improvement approaches in enhancing emergency care.

## Introduction

A pneumothorax is a collection of air outside the lung but within the pleural cavity. It occurs when air accumulates between the parietal and visceral pleura inside the chest. Air accumulation can apply pressure to the lungs and cause them to collapse. Pneumothoraces can be further classified as simple, tension, or open. A simple pneumothorax does not shift the mediastinal structures, but a tension pneumothorax does [1-2]. The management in most of the cases is chest drain and sometimes needle aspiration [3-4]. Tension pneumothorax is a life-threatening condition that requires immediate intervention, yet delays in accessing chest drain equipment in the Emergency Department (ED) pose significant risks to patient safety [5].

A reported incident in our organization highlighted the risks of procedural delays; specifically, there was a documented delay in inserting a chest drain for a life-threatening pneumothorax, which was recorded via Datix. Second, evidence from best practices showed that the introduction of procedure-specific trolleys, similar to those used for fascia iliac block procedures, had previously improved efficiency by centralizing necessary equipment. Finally, a previous quality improvement project (QIP) on the respiratory ward, which focused on chest drains, had yielded positive results, further supporting the potential for similar strategies to improve efficiency and patient safety. These findings emphasized the need to streamline the chest drain insertion process.

The objectives of the project were to enhance the ease and efficiency of gathering the necessary equipment for chest drain insertion. Specifically, the project aimed to improve patient safety by ensuring timely and efficient chest drain insertions, which can prevent death and serious complications in patients with life-threatening pneumothorax or haemothorax. It also sought to optimize the ED workflow by reducing the time required to collect and prepare equipment, allowing clinicians to focus on patient assessment and other critical tasks. Additionally, the project aimed to enhance patient outcomes by supporting faster interventions and reducing delays.

This QIP aimed to address these issues by implementing a dedicated chest drain trolley, ensuring all necessary items were readily available and organized in the ED resuscitation area. Using the methodology, the project focused on reducing delays, enhancing staff confidence, and improving patient outcomes, offering a structured approach to tackling critical emergency care challenges.

## Technical report

**Figure 1:**
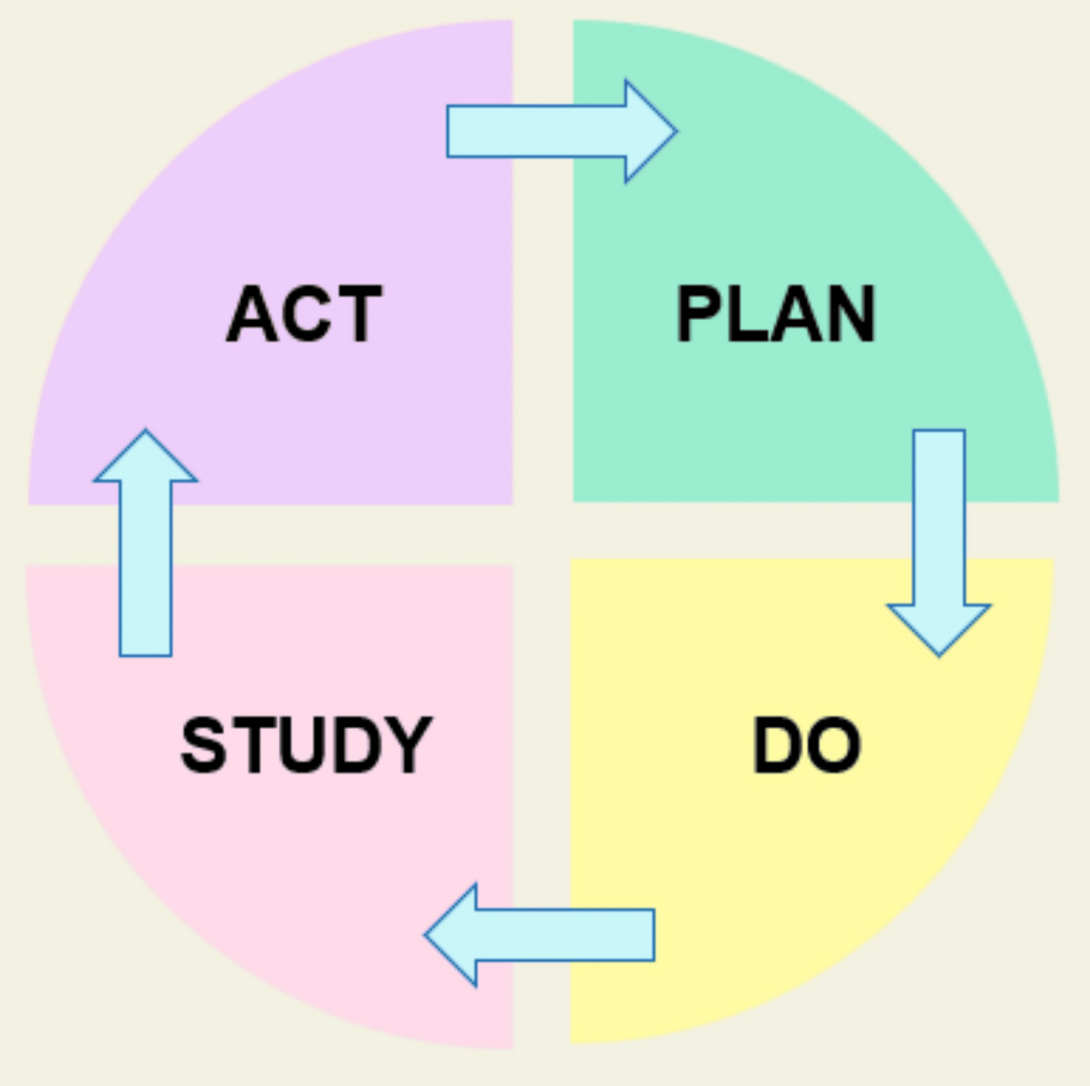
Plan-Do-Study-Act (PDSA) cycle for the quality improvement project

The quality improvement methodology employed was the Plan-Do-Study-Act (PDSA) cycle (Figure 1), which allowed for iterative testing and refinement of solutions. This approach was selected for its practicality in identifying problems, implementing changes, and evaluating outcomes in real time. In the Plan phase, the initial steps of the project included team formation, where a multidisciplinary team consisting of doctors, nurses, and healthcare assistants (HCAs) was assembled, with leadership from the project initiator and guidance from an ED registrar. 

**Figure 2:**
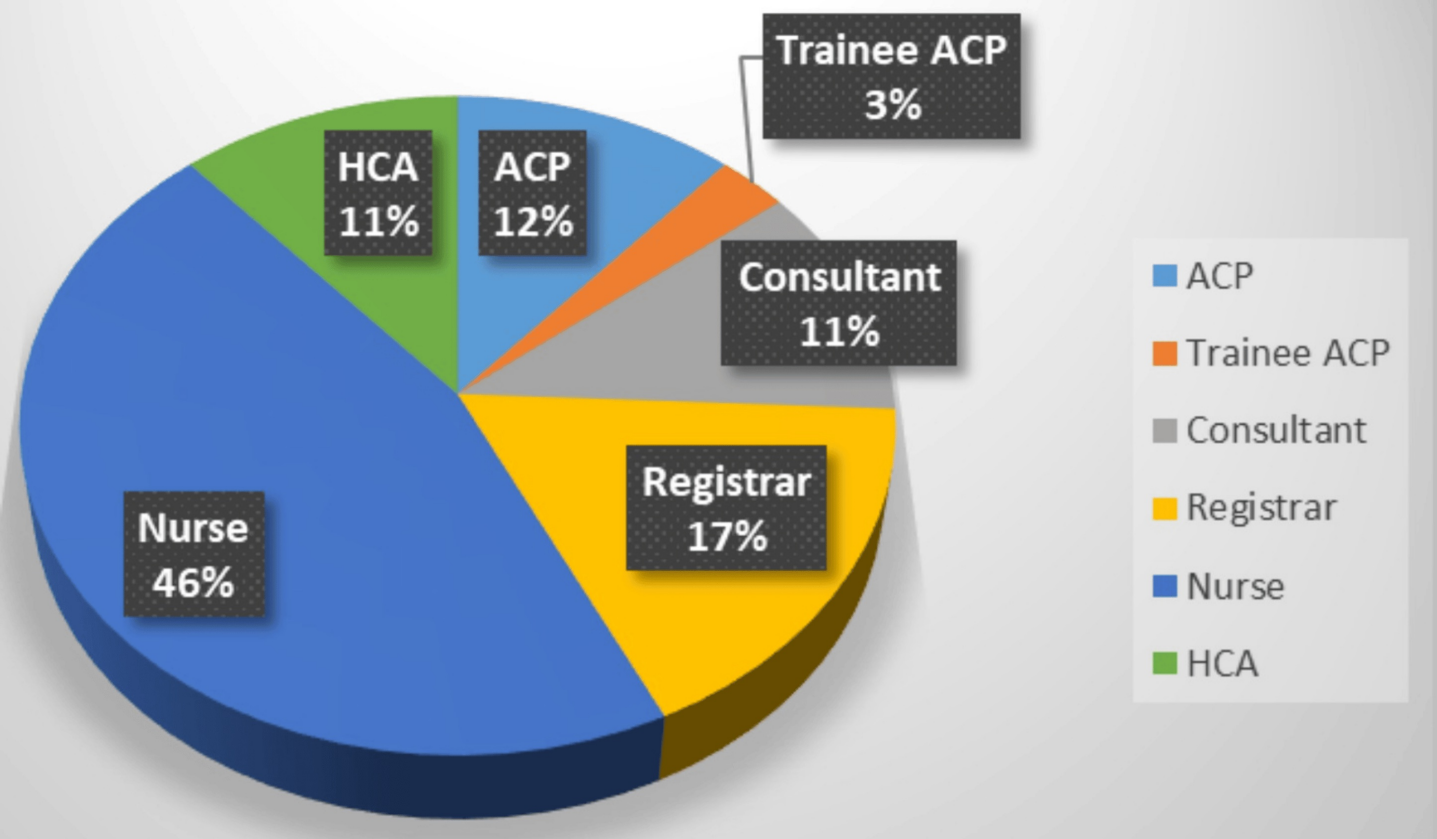
Participants in the survey ACPs: advanced clinical practitioners; HCAs: healthcare assistants

A baseline survey was conducted to gather initial data on current practices and perspectives related to the process of preparing for chest drain insertion. The first survey was distributed to doctors, advanced clinical practitioners (ACPs), and physician associates (PAs) between 23 January 2024 and 4 February 2024. These clinicians were selected first because they are typically responsible for performing the procedure itself. Following this, the same two survey questions were sent to nurses and HCAs between 9 February 2024 and 12 February 2024. These staff members often play a key role in gathering the necessary equipment and supporting the procedure itself. This approach ensured that feedback was collected from all relevant groups involved in the chest drain insertion process, providing a comprehensive understanding of current challenges and opportunities for improvement (Figure 2).

**Figure 3:**
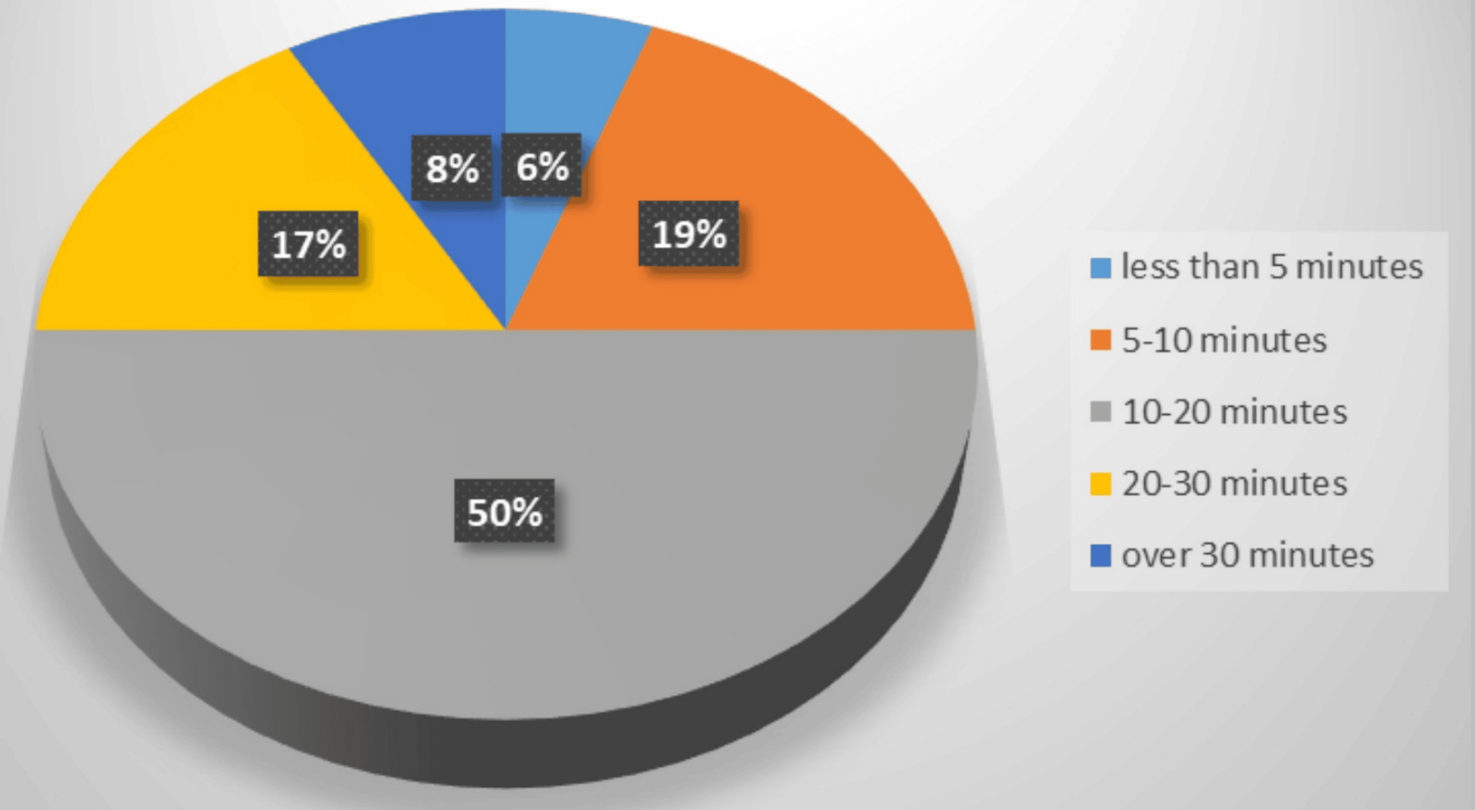
Pre-intervention survey Q1: roughly how long does it take to gather the equipment required for chest tube insertion? The first survey revealed that 50% of the targeted healthcare providers were able to gather the equipment within 20 minutes, while up to 25% took longer

**Figure 4:**
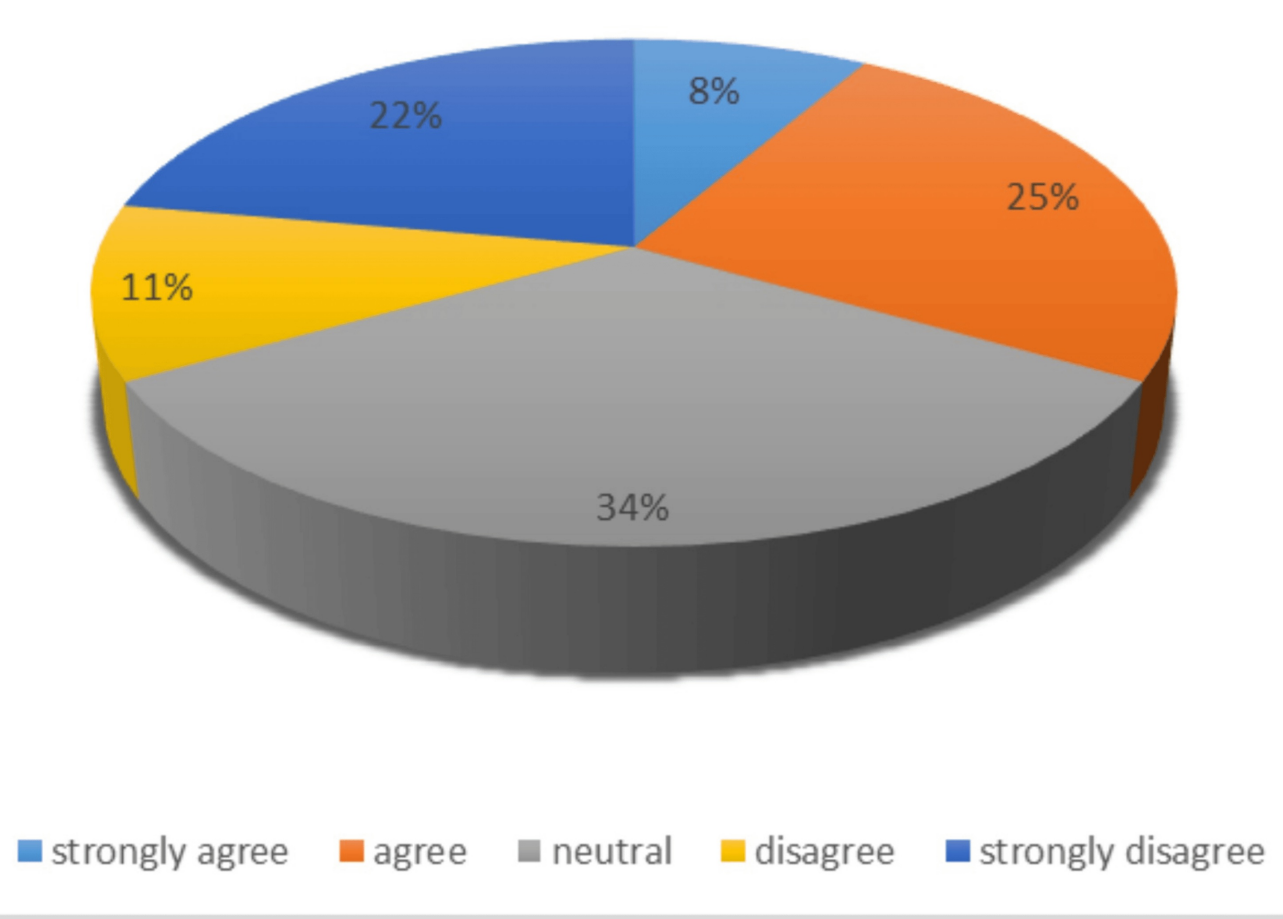
(Q2) How strongly do you agree or disagree with the following statement: 'I know where to find all the equipment required to insert a chest drain'? The first survey showed that more than 30% of the staff were unaware of the locations of the required equipment, and more than 30% were not confident

The survey consisted of two primary questions, with responses serving as the foundation for future actions. Key findings highlighted notable inefficiencies: Most respondents reported excessive time required to gather equipment and a lack of awareness about the equipment’s exact location due to the multiple storage areas where the equipment is kept. Proposed improvements included consolidating all necessary items in a dedicated trolley or organized drawers, introducing a checklist for regular stock maintenance, and providing targeted training to familiarize staff with the required equipment. These insights emphasized the critical need for streamlined processes and improved organization to enhance efficiency (Figures 3-4).

**Figure 5:**
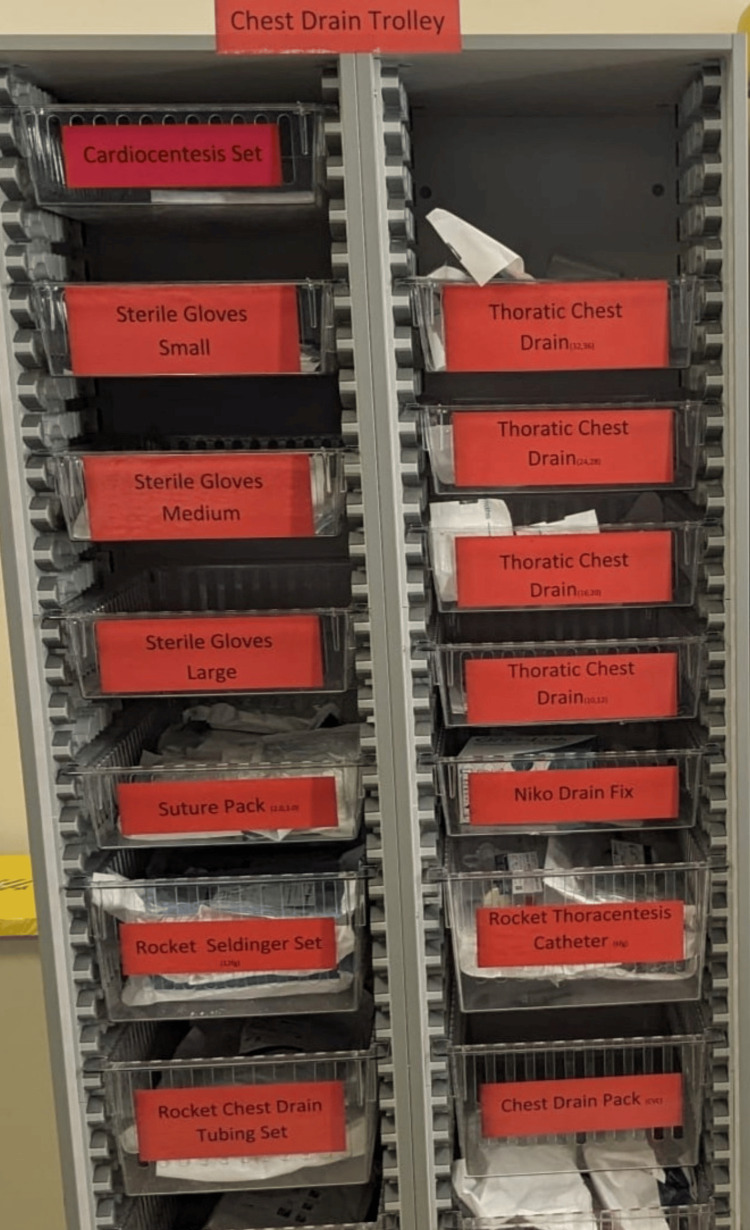
A chest drain trolley was installed in the resuscitation area

In the Do phase of the PDSA cycle, the project team implemented key actions to support the organization of chest drain equipment drawers and training simulation sessions. To achieve this, the team engaged relevant stakeholders and collaborated with the financial department to secure funding for a large trolley with drawers, essential for the project's objectives. After providing a clear explanation of the project's purpose, objectives, and anticipated benefits, the financial department approved the purchase, and it was installed (Figure 5). This trolley would enable efficient stocking through the use of a checklist while also supporting improved access to chest drain equipment for training simulation sessions. The checklist was also created to make sure that it will be well stocked (Table 1). The successful engagement with stakeholders and acquisition of resources marks an important milestone in advancing the project's goals.

**Table 1:** Checklist for stocking the chest drain trolley

	Date: ………………..	Date: ………………..	Date: ………………..	Date: ………………..
	Checked by: ………………..	Checked by: ………………..	Checked by: ………………..	Checked by: ………………..
Chest drain pack				
Sterile gowns				
Rocket thoracentesis catheter				
Rocket seldinger set				
Rocket chest drain tubing set				
Thoracic chest drain (32,36)				
Thoracic chest drain (24, 28)				
Thoracic chest drain (16, 20)				
Thoracic chest drain (10, 12)				
Bottle				
Niko drain fix				
Sterile gloves small/medium/large-sufficient amount Y/N				
Suture pack				

In the Study phase of the QIP, the focus was on identifying the root causes of inefficiencies in accessing and utilizing the chest drain trolley in the ED. Simulation round one was conducted on 10 April 2024 using a mannequin to replicate real-life scenarios. It took approximately seven minutes to gather the necessary equipment during this simulation. Key issues identified included poor labeling, inaccessible sterile water, and confusion regarding chest drain kits. Additionally, the simulation revealed that not all staff were aware of the trolley’s location or its contents, contributing to delays.

**Figure 6:**
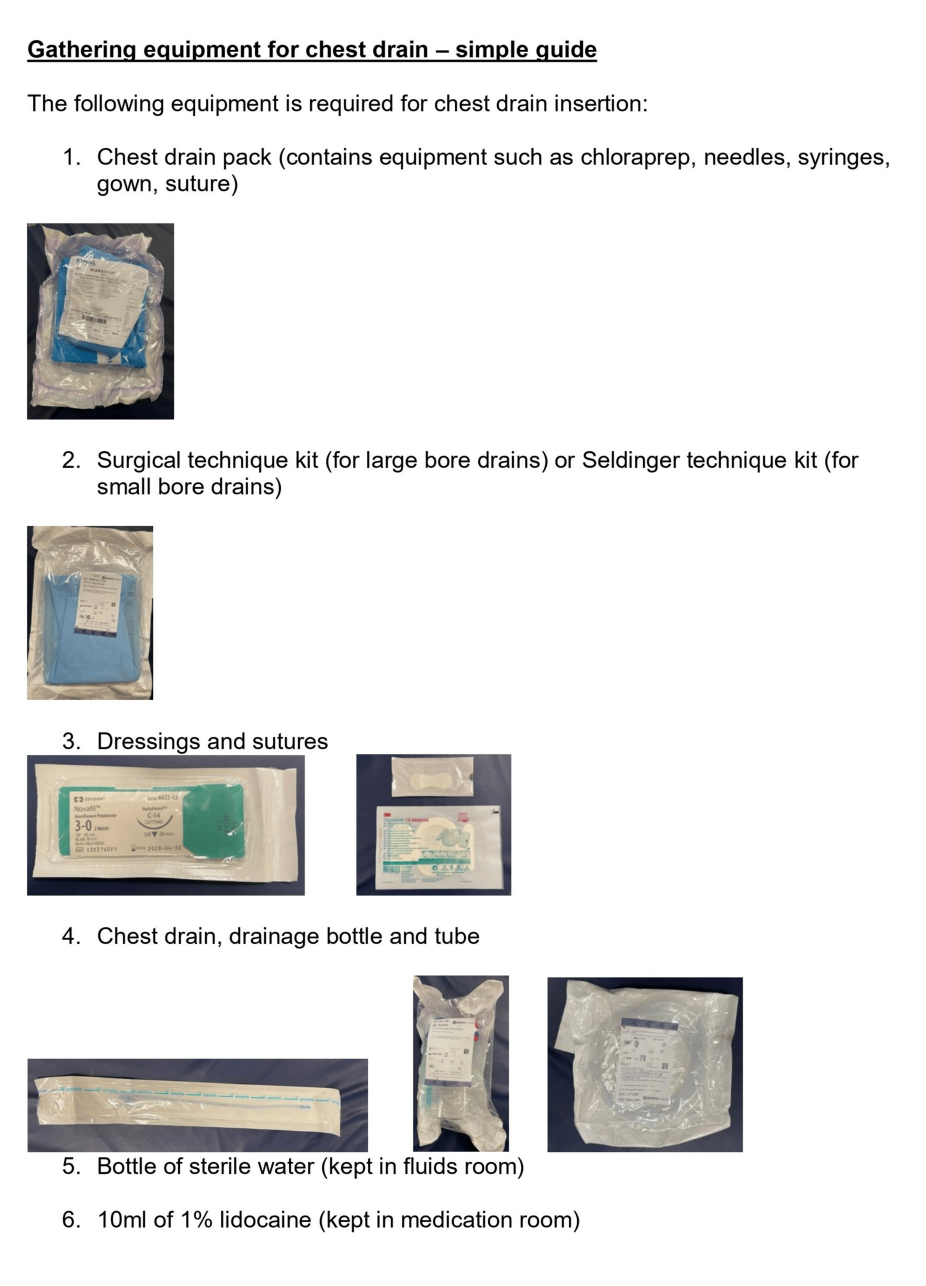
Checklist for the procedure's items ED: Emergency Department Pictures of the kits were attached to the checklist to increase awareness of all the items. Given the busy environment in the ED, any healthcare professional, regardless of their background knowledge or familiarity with the hospital, may be asked to prepare the chest drain. Thanks to the checklist, they will be able to do that with no problem. The list contains the following: 1-gown, 2-chest drain kit (containing all the necessary syringes that will be used during the procedure), 3-dressing and sutures, 4-chest drain bottle and a connecting tube, 5-bottle of water, 6-lidocaine

Addressing these issues was deemed essential to streamline the preparation process and reduce unnecessary delays. To ensure staff awareness of the chest drain trolley, an email was sent to all staff members across the organization, regardless of their role, providing information about the trolley along with a picture and a map indicating its location. To prevent errors among newly joined or junior staff, a checklist for the procedure was developed, featuring six clear items with visual aids to enhance accessibility (Figure 6). Following these interventions, a second simulation was conducted to evaluate the effectiveness of the implemented changes.

During Simulation 2 on 22 April 2024, a different team, primarily composed of staff from the medical team located in the ED, participated in the assessment. They recorded a time of three minutes to gather equipment for a chest drain insertion, demonstrating significant improvement compared to the first simulation. Key changes implemented included placing sterile water in the trolley after pharmacy approval that it does not need to be locked and using the checklist to ensure all necessary items were collected. Despite these improvements, some issues persisted. The procedure table remained unavailable due to delayed procurement, junior staff from the acute medicine team were untrained, and both lidocaine and dressings were forgotten until after the procedure had begun, even though the checklist was positioned on the side of the trolley.

**Figure 7:**
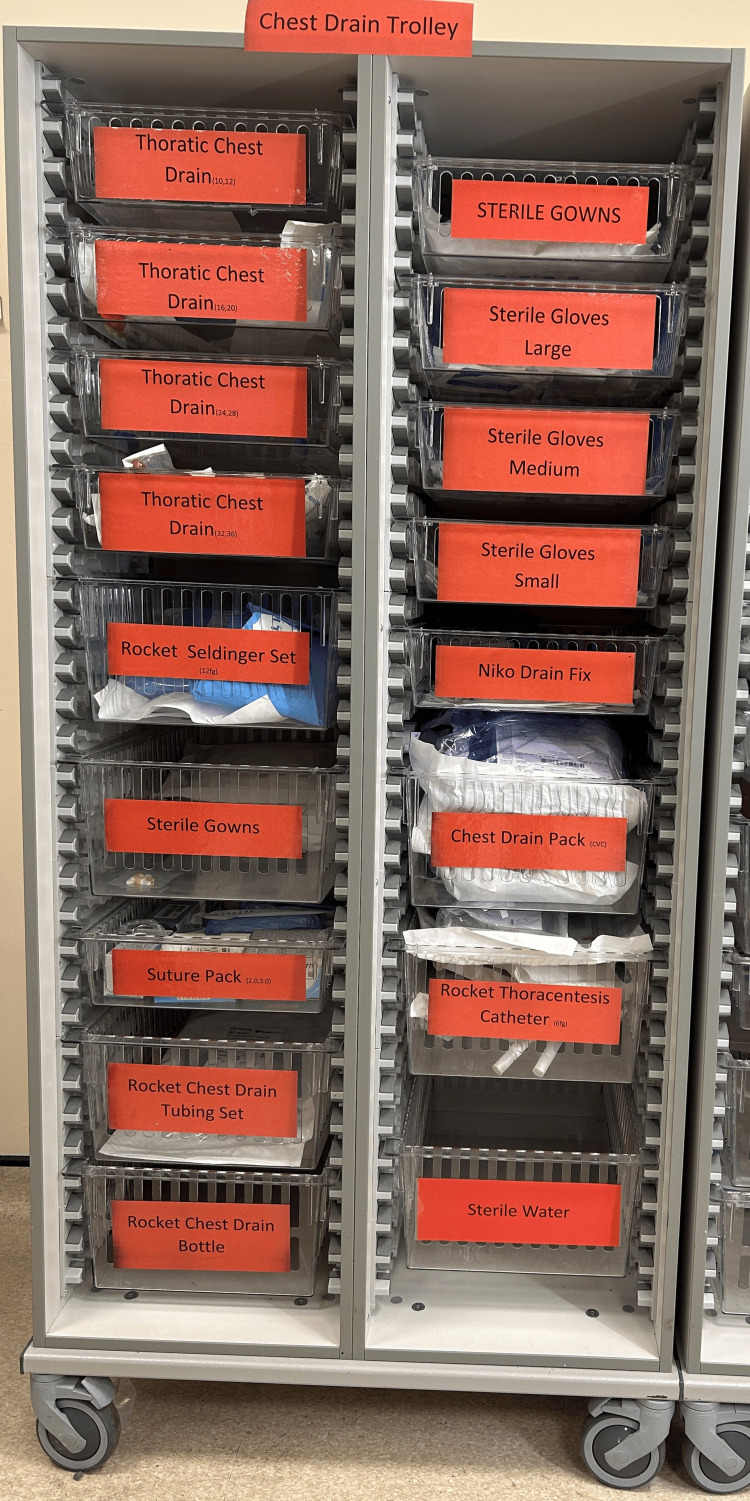
Chest drain trolley rearranged in proper order

To address these challenges, the team proposed expediting the procurement of the procedure trolley, maintaining the checklist but repositioning it at the front of the trolley to ensure all necessary items are accounted for, and organizing and numbering the trolley drawers (from 1 to 6) in accordance with the procedural steps to enhance efficiency and preparedness (Figure 7). This revised approach aims to improve overall readiness and minimize errors during future simulations.

During the Act phase of the PDSA cycle, the team assessed the impact of the implemented changes and identified further steps to sustain progress. Repeat survey results indicated improvements in clinical practice, demonstrating the effectiveness of the changes, such as the revised equipment organization and checklist implementation. However, ongoing assessment highlighted opportunities for additional refinements, such as enhanced training for junior staff and continued evaluation of the procedure trolley and equipment accessibility. These findings will guide the next cycle of adjustments to ensure sustained improvements in efficiency, coordination, and preparedness for chest drain insertion procedures.

**Figure 8:**
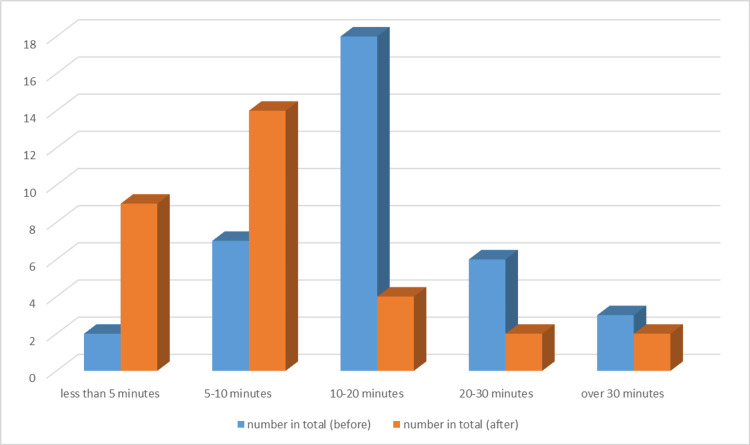
Pre- and post-intervention survey Q1: roughly how long does it take to gather the equipment required for a chest drain procedure? QIP: quality improvement project A remarkable increase was observed in the number of healthcare professionals able to efficiently collect items for chest drain procedures following the completion of the QIP

**Figure 9:**
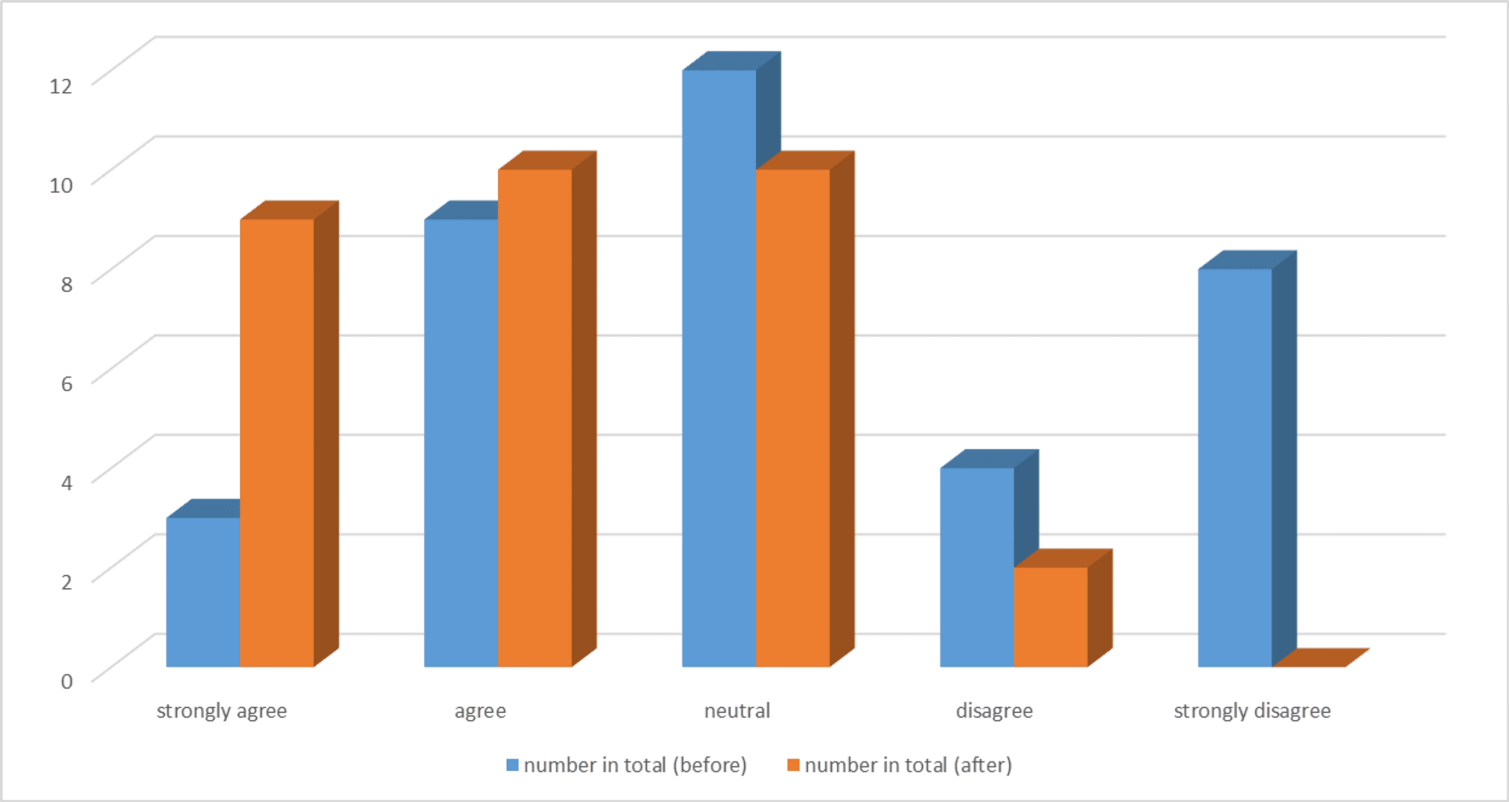
Pre- and post-intervention survey Q2: how strongly do you agree or disagree with the following statement: 'I know where to find all the equipment required to insert a chest drain'? QIP: quality improvement project A significant increase in the number of individuals now aware of the chest drain trolley after the QIP is noticeable (represented in orange)

**Figure 10:**
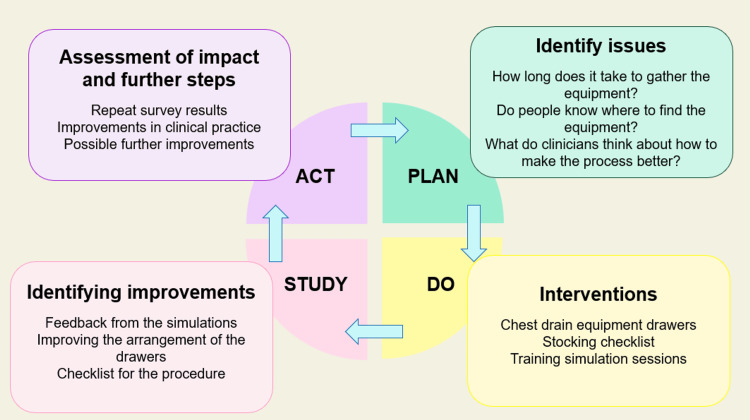
Plan-Do-Study-Act (PDSA) completed cycle

A follow-up survey in August 2024 indicated that over 90% of the staff were aware of the trolley’s location and contents, with preparation times consistently reduced to under five minutes. The feedback on the organization and checklist use was overwhelmingly positive (Figures 8-9). Interventions included improved chest drain equipment drawer organization by nursing staff, prioritized weekly equipment checks, and simulation training sessions that addressed procedural inefficiencies and ensured all staff were familiar with the process. By finishing this last step, we were able to finish the PDSA cycle for our quality improvement with room for improvement for the next QIP if needed (Figure 10). 

## Discussion

Effective and timely management of life-threatening conditions such as pneumothorax is critical, as delays in chest drain insertion can lead to severe complications, including respiratory failure and death [6-7]. The implementation of the chest drain trolley system ensures that essential equipment is readily available, reducing delays in chest drain insertion and enabling clinicians to perform life-saving interventions more efficiently.

The QIP effectively identified critical inefficiencies in the chest drain insertion process within the ED through the analysis of performance data gathered from surveys and direct observations. Key findings revealed an average preparation time of 20 minutes and a significant gap, with 60% of staff unaware of equipment locations. A pre-intervention survey conducted between January and February 2024 assessed 25 ED healthcare professionals’ familiarity with chest drain equipment, procedural readiness, and knowledge of equipment storage locations. The survey highlighted issues such as scattered equipment storage, lack of awareness regarding equipment placement, and a need for a dedicated trolley equipped with a clear checklist.

To address these inefficiencies, a PDSA cycle was initiated. Between February and August 2024, iterative improvements were made to refine the process. In April and May 2024, two simulation sessions took place following the setup of a preliminary trolley. Key challenges, such as poor drawer organization and inaccessible sterile water, were addressed through refinements like drawer labeling, in collaboration with the pharmacy to ensure that sterile water remained accessible, and the introduction of a new checklist. These changes resulted in a dramatic reduction in preparation time from 20 minutes initially to seven minutes following the first simulation, and just three minutes after the second simulation on 22 April 2024.

A second survey distributed in August 2024 reassessed staff satisfaction, confidence, and perceptions of the implemented changes. The feedback confirmed the trolley’s positive impact on reducing delays, boosting staff confidence, and improving equipment accessibility. However, ongoing challenges such as untrained junior staff and occasional missing items remain, highlighting the need for continued testing, staff engagement, and sustained buy-in to drive further improvements. Numerous studies have emphasized the need for healthcare organizations to continue quality improvement initiatives related to chest drain procedures, as they can have a profound impact on patient safety [8].

## Conclusions

The implementation of the chest drain trolley system, supported by targeted staff training and simulation exercises, resulted in significant improvements in the efficiency and safety of chest drain insertion procedures within the ED. Key learning points from this QIP emphasize the importance of multidisciplinary collaboration, the role of real-time simulation in identifying and addressing procedural gaps, and the critical role of systematic equipment management through checklists and organized storage.

The QIP demonstrates the potential for similar initiatives to enhance workflow efficiency, reduce procedural risks, and improve patient outcomes in emergency medical settings. Continued emphasis on the involvement of all relevant staff groups, maintenance of equipment availability and accessibility, and the use of simulation training for real-time process refinement is essential to sustain these improvements. Ongoing testing, staff engagement, and continuous assessment are crucial for optimizing workflows and ensuring long-term success in clinical practice enhancement.
